# Enhanced performance of gene expression predictive models with protein-mediated spatial chromatin interactions

**DOI:** 10.1038/s41598-023-38865-5

**Published:** 2023-07-20

**Authors:** Mateusz Chiliński, Jakub Lipiński, Abhishek Agarwal, Yijun Ruan, Dariusz Plewczynski

**Affiliations:** 1 Laboratory of Bioinformatics and Computational Genomics, Faculty of Mathematics and Information Science, Warsaw University of Technology, 00-662 Warsaw, Poland; 2 Laboratory of Functional and Structural Genomics, Centre of New Technologies, University of Warsaw, 02-097 Warsaw, Poland; 3 Cellular Genomics, Warsaw, Poland; 4 The Jackson Laboratory for Genomic Medicine, 10 Discovery Drive, Farmington, CT 06030 USA; 5 Life Sciences Institute, Zhejiang University, Zhejiang, Hangzhou China

**Keywords:** Computational biology and bioinformatics, Genetics, Molecular biology, Systems biology, Molecular medicine

## Abstract

There have been multiple attempts to predict the expression of the genes based on the sequence, epigenetics, and various other factors. To improve those predictions, we have decided to investigate adding protein-specific 3D interactions that play a significant role in the condensation of the chromatin structure in the cell nucleus. To achieve this, we have used the architecture of one of the state-of-the-art algorithms, ExPecto, and investigated the changes in the model metrics upon adding the spatially relevant data. We have used ChIA-PET interactions that are mediated by cohesin (24 cell lines), CTCF (4 cell lines), and RNAPOL2 (4 cell lines). As the output of the study, we have developed the Spatial Gene Expression (SpEx) algorithm that shows statistically significant improvements in most cell lines. We have compared ourselves to the baseline ExPecto model, which obtained a 0.82 Spearman's rank correlation coefficient (SCC) score, and 0.85, which is reported by newer Enformer were able to obtain the average correlation score of 0.83. However, in some cases (e.g. RNAPOL2 on GM12878), our improvement reached 0.04, and in some cases (e.g. RNAPOL2 on H1), we reached an SCC of 0.86.

## Introduction

**Figure 1:**
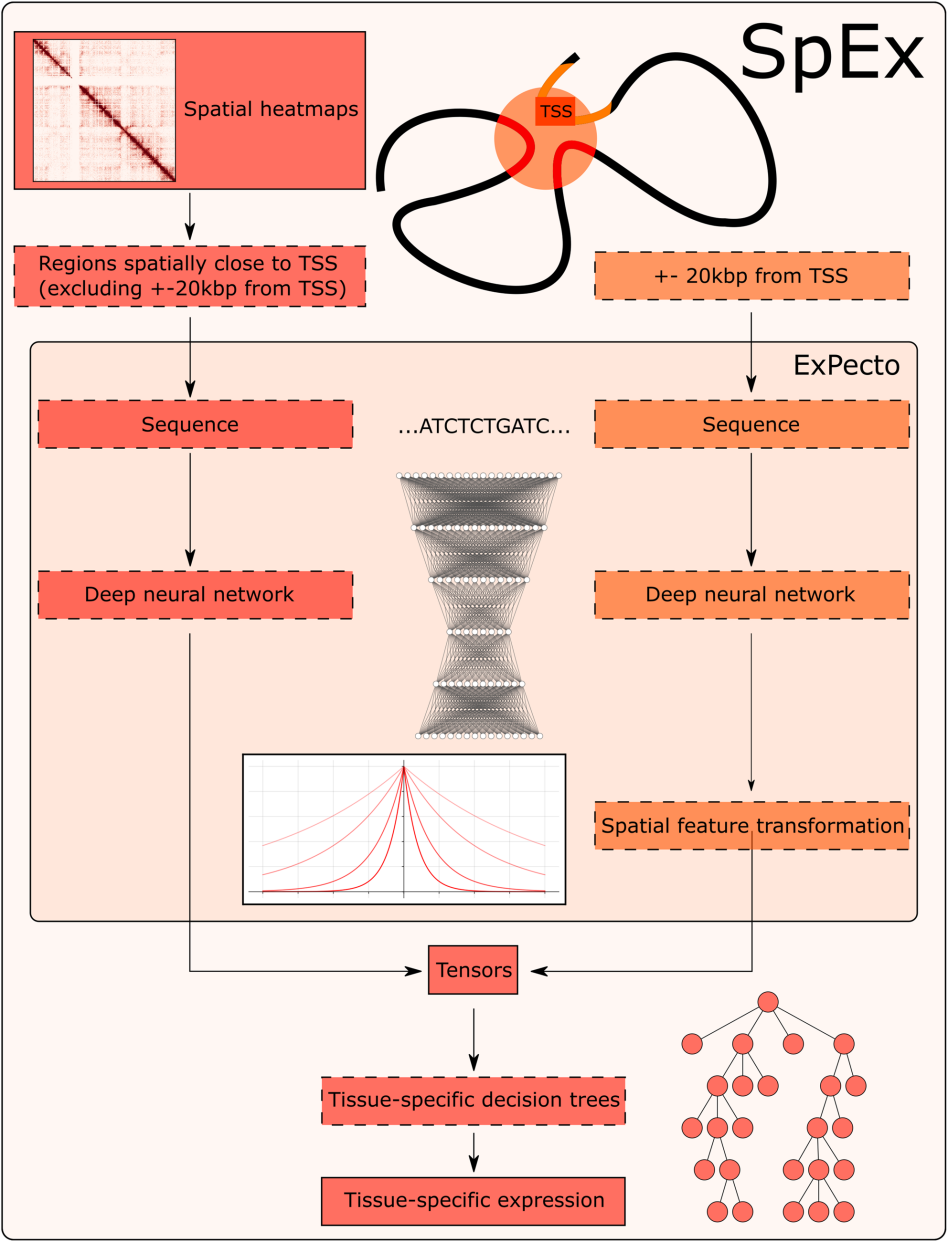
The architecture of SpEx. The spatial heatmaps are used for obtaining the regions close to the TSS (excluding +—20 kbp from TSS), and sequence from those regions is taken, and put into classic deep learning ExPecto model—which generates epigenetic signal over those regions. The classical features from ExPecto are merged with those obtained from spatially close regions, and the decision trees predict the expression levels. See “Methods” for more information about the algorithm.

The advances in the field of Machine Learning have revolutionised other fields as well. With the increasing computational power and decreasing costs, the predictive power of modern-day deep learning networks allows scientists to apply those methods to various tasks that would be impossible to solve otherwise. Those advances did not omit the genomics field as well^1,2^. The first attempts to predict the expression solely on the DNA sequence started just after The Human Genome Project^3^—however, they had a vast number of limitations^4,5^ and have mainly concentrated on the classical modelling approaches. However, those limitations started to disappear with the expansion of deep learning models. One of the first major studies on the usage of CNNs^6^ and XGBoost^7^ started a new era in predicting the expression with the introduction of ExPecto^1^. Then it continued with the use of CNNs through multiple models, including Basenji2^8^, and finally with the use of transformer-based models like Enformer^2^. However, in our study, we have decided to take a standard approach available with the help of CNNs and expand it further with the input change to include spatial genomic information. The ExPecto model we decided to advance takes 20kbp surrounding the TSS of a given gene and uses expression from that to train a deep neural network to predict the epigenetic factors. Using those factors the tissue-specific gene expression profile is calculated with a high Spearman correlation score. In our study, we have investigated if the epigenetics marks alone are sufficient for the complex task of prediction of the expression—and have given a hypothesis that while they are incredibly informative, there is still a place for improvement. We decided that we would like to investigate the effects of the spatial chromatin architecture inside cell nuclei on the expression by exploring the models created with 3D information available and without it. To do that, we have modified the ExPecto algorithm accordingly, so it uses not only the 20kbp region around the TSS but also regions that are linearly distal—but are, in fact, spatially close, thanks to the spatial interactions that are mediated by specific proteins of interest. The overview of the algorithm proposed by us, SpEx (Spatial Gene Expression), is shown in Fig. 1.

To prove the model's validity, we decided to create an empirical study on how specific protein-mediated interactions are helping in the prediction of gene expression. To do that, we have selected the three most important proteins for loop creation—cohesin, CTCF, and RNAPOL2. The effects of those proteins being unable to bind or be created properly were shown in multiple studies and were the inspiration for asking whether the machine learning models, provided we add 3D information (from interactions mediated by those proteins), will improve.

### Proteins of interest

Cohesin is a protein complex discovered in 1997^9,10^ by two separate groups of scientists. The complex is made out of SMC1, SMC3, RAD21, and SCC3. However, in human cell lines, SCC3 (present in yeast) is replaced by its paralogues—SA1^11^, SA2^12^, and SA3^13^. However, SA3 appears only in cohesin during mitosis^14^, and we will concentrate on SA1 and SA2 since they are forming cohesin in somatic cells. The complex is essential in the proper functioning of the cell nucleus—as is fundamental for the loop extrusion^15^, it stabilises the topologically associating domains (cohesin-SA1)^16^, allows interactions between enhancers and promoters (cohesin-SA2)^16^. The depletion of cohesin in a nucleus removes all the domains^17^, and completely destroys the spatial organisation of the chromatin. Mutations of cohesin negatively affect the expression of the genes—e.g. in Cornelia de Lange syndrome^18,19^ and cancer^20^, where the altered complex is incapable of sustaining its proper function, leading to diseases.

CTCF (CCCTC-binding factor) is an 11-zinc finger protein. Its primary function is the organisation of the 3D landscape of the genome^21^. This regulation includes: creating topologically associated domains (TADs)^22–24^, loop extrusion^25^, and alternative splicing^26^. The protein very often works with the previously mentioned cohesin complex, allowing loop formation. CTCF, as a regulator of the genome, binds to specific binding motifs and regulates around that loci. That is why, in case of mutations in the motifs, it might bind improperly, thus allowing disease development. However, not only mutations in the binding sites are disease prone. Mutations in the CTCF protein itself have proven to significantly influence the development of multiple conditions. Some of the examples of diseases induced by a mutation in the CTCF proteins include MSI-positive endometrial cancers^27^, breast cancers^28,29^, and head or neck cancer^30^.

Therere are three common RNA Polymerase complex proteins in eukaryotic organisms—I, II, and III^31^. In this study, we will focus mainly on RNAPOL2, as that is responsible for the transcription of the DNA into messenger RNA^32,33^, thus having the most significant impact on the expression of the genes. The mechanisms responsible for creating the RNAPOL2 loops are complex and require not only RNAPOL2 protein but also several other transcription factors^34,35^. The mutations in those transcription factors have been shown to be linked to various diseases^36^, including acute myeloid leukaemia^37–39^, Von Hippel–Lindau disease^40,41^, sporadic cerebellar hemangioblastomas^42^, benign mesenchymal tumours^43^, xeroderma pigmentosum, Cockayne syndrome, trichothiodystrophy^44^, and Rubenstein-Taybi syndrome^45^.

### Protein-mediated interactions

Multiple studies have shown the spatial landscape created by cohesin-mediated chromatin loops. The first major cohesin ChIA-PET study from 2014^46^ showed the internal organisation of chromatin in the chromosomes. For example, the study provided a list of enhancer-promoter interactions, which can be a starting point for gene expression study.

The next study from 2020^47^ extended the 2014 study and showed that among 24 human cell types, 72% of those loops are the same; however, the remaining 28% are correlated to the gene expression in different cell lines. Those loops mostly connect enhancers to the promoters, thus regulating the gene expression. Another interesting insight from this study is that those different profiles of interactions are effective in clustering the cell types depending on the tissue they were taken from.

CTCF, as mentioned above, is responsible for loop extrusion. That is why it is very popular to investigate CTCF-mediated interactions. Once again, like with the cohesin complexes, ChIA-PET is used for obtaining the interactions mediated by CTCF. One of the major studies from 2015^48^ shows the genomic landscape among 4 cell lines. They discovered that SNPs occurring in the motif of the CTCF-binding site can alter the existence of the loop—and by that, contribute towards the disease development. They assessed the SNPs residing in the core CTCF motifs and found 70 of those SNPs. Of those, 32 were available from the previously done GWAS studies, and 8 were strongly associated (via linkage disequilibrium) with disease development.

Another study from 2019^49^ analysed mutations using 1962 WGS data with 21 different cancer types. Such an analysis, enhanced with the usage of CTCF ChIA-PET data, showed that disruptions of the insulators (that are creating the domains) by motif mutations and improper binding of CTCF (and, by that, diminish of the loop) lead to cancer development. Using a computational approach, they have found 21 potentially cancerous insulators.

The transcription chromatin interactions, such as the ones mediated by RNAPOL2, are of great interest as well—they control the transcription directly, after all. The study from 2012^50^ showed the RNAPOL2-mediated ChIA-PET interactions on 5 different cell lines to show the transcriptional genomic landscape. Another study from 2020^51^ performed the same experiments on RWPE-1, LNCaP, VCaP, and DU145 cancer cell lines. Similar to the 2012 study, they have shown the spatial interactions based on RNAPOL2, but this time in cancer cell lines. Furthermore, they showed that cohesin and CTCF interactions provide a stable structural framework for the RNAPOL2 interactions to regulate the expression, thus making all of the proteins that we describe in this section crucial for the proper expression of the genes.

Those findings were the main motivation for our analysis—as based on the evidence, the cohesin, CTCF, and RNAPOL2 interactions should give us more information on the genetic expression, thus improving the metrics for the machine learning models. In this work, we present an extension of the ExPecto^1^ deep learning model that is enriched with spatial information, thus, as expected, improving the statistical metrics.

### ExPecto architecture

ExPecto^1^ is a model introduced in 2018 for predicting gene expression from the sequence. It uses a deep neural network (namely, Convolutional Neural Network—CNN). It is composed of, most importantly, 6 convolutional layers, 2 MaxPoolings (the activation function for all the layers is ReLU). For the exact architecture, see the original paper. As mentioned, the input to the network is the DNA sequence, and the output is in the form of the 2002 epigenetic factors—collected from ENCODE and Roadmap Epigenomics. The network takes 2000 bp as the window and predicts the epigenomic of its 200 bp middle, using the remaining base pairs as the context. The model is then applied to 20,000 bp region surrounding TSS, and the step size is determined by the aforementioned 200 bp, yielding 2002 features multiplied by 200 bins (100 left and 100 right), so the total number of features describing the gene is 400,400. Then, those features are transformed using exponential functions (10 upstream and 10 downstream TSS), so the final number of the features is 40,040. Then, xgboost (namely, gradient boosting of linear regression models) is used for the prediction of the expression of gene expression. They obtained a Spearman correlation score of 0.819, and the testing was done on chromosome 8.

## Results

**Figure 2:**
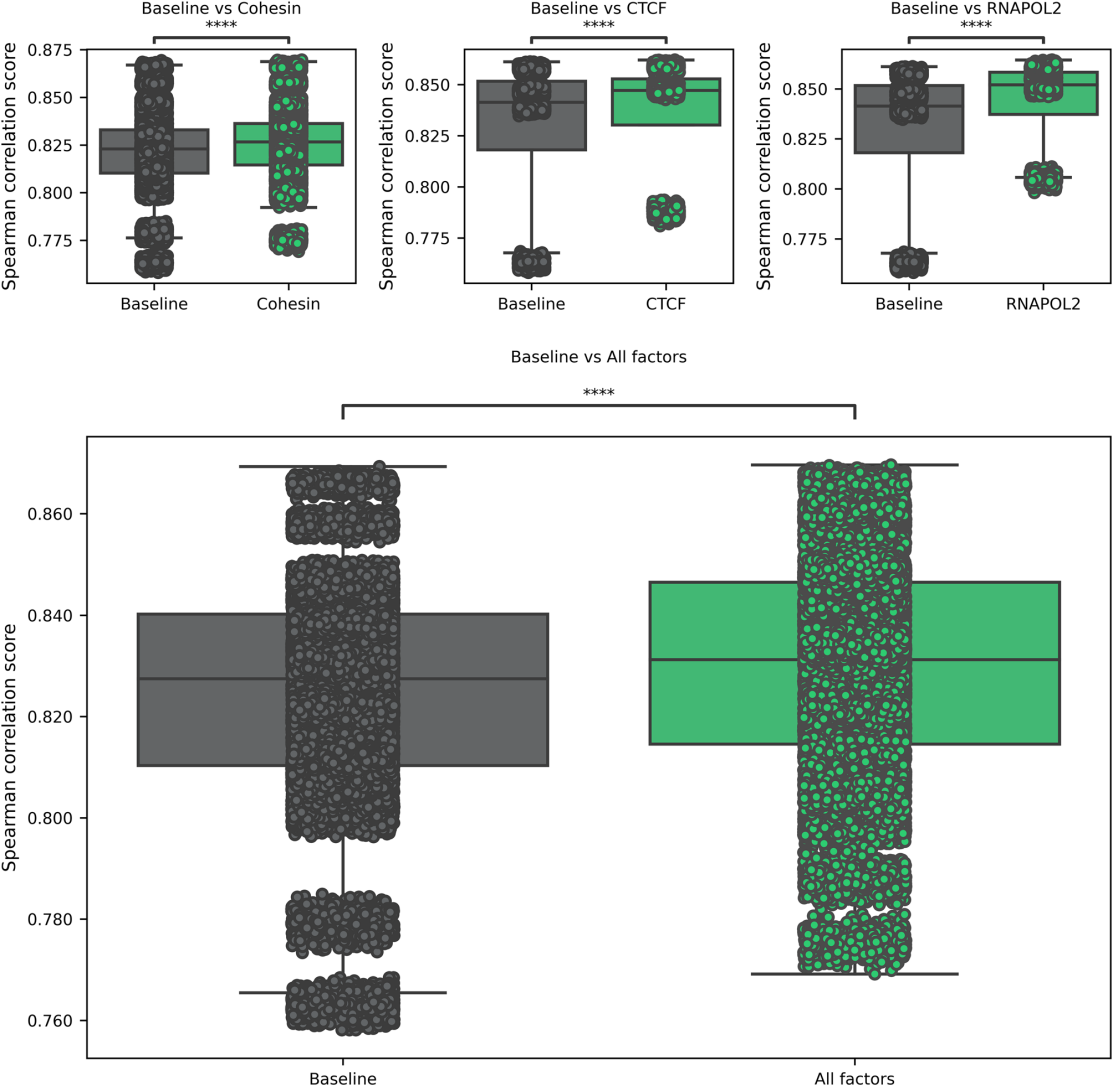
Statistical analysis of the Spearman correlation score between the baselines and the experiments grouped by the factor of interest (cohesin, CTCF, RNAPOL2). See Supplementary Table 1 for details on which experiments are included in the specific factor group.

To study those changes, we have gathered 24 cell lines for the cohesin ChIA-PET and 4 cell lines for CTCF and RNAPOL2 binding factors^52,53^. They were all mapped to the closest tissue with available gene expression profile from the connected GTEx^54^, ENCODE^55^, and Roadmap epigenomics^56^ database released by ExPecto authors. The model's training was performed 1000 times to ensure the statistical significance of the findings. To compare the best with other models (ExPecto, Enformer), we have focused on Spearman's rank correlation coefficient (SCC). However, the analysis was repeated for the Pearson correlation coefficient and root-mean-square error (RMSE). The results of that analysis were similar to the ones performed using SCC, and details about it can be found in Supplementary Figs. 3–6. The results for each experiment in the case of SCC can be seen in Supplementary Fig. 1. The greatest improvements in the Spearman correlation score can be seen in the models that use heatmaps from RNAPOL2 ChIA-PETs. In that case, the metric's improvement was up to even 0.042 (in RNAPOL2 ChIA-PET GM12878), and the average improvement was 0.016. In the case of CTCF, the greatest improvement was also in GM12878, with an improvement of 0.025, with the average improvement over the CTCF study of 0.009. In the case of the cohesin ChIA-PETs, the highest improvement was seen in the K562 cell line, as it totalled 0.020, with an average increase of the correlation score of 0.004. Furthermore, all of the tests were found to be statistically significant, with all the p-values < 10e−11, with the exception of two tests: cohesin ChIA-PET KU19, which obtained a p-value of 0.000103, and cohesin ChIA-PET H1, which obtained p-value of 0.01014. The average improvement over the whole dataset was established at 0.0058 (0.007 for Pearson correlation coefficient, and around 2% improvement over RMSE), and all the grouped sets (cohesin, CTCF, RNAPOL2) were statistically significant at p-value < 10e−31. The cumulative results can be seen in Fig. 2.

**Figure 3:**
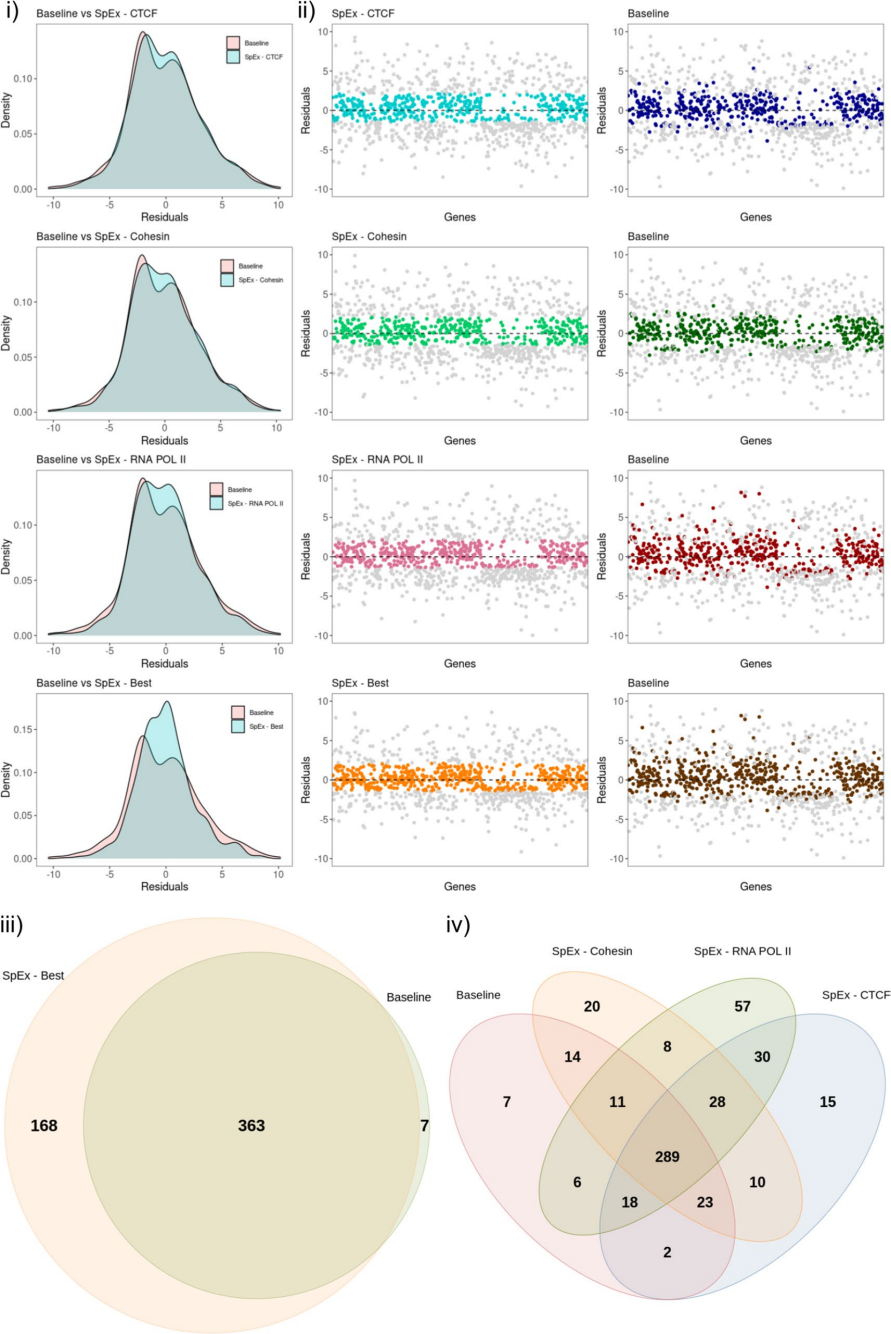
(**i**) Distribution of residuals for all the protein factors and SpEx-Best, along with the comparison of residual of baseline. (**ii**) Scatter plot of all genes (n = 990) with respect to their residual value; highlighted genes are within cutoff (− 1.397/+ 2.106 of SpEx), same genes mapped on the baseline. (**iii**) Venn Diagram of the genes within cutoff (n = 538) that are improved by SpEx best in comparision of the baseline. (**iv**) Venn diagram of genes within cutoff (n = 538) that are improved by SpEX for each protein factor in comparision with the baseline.

Further, to investigate the model in more detail, we compared the residuals of the baseline model with the ones obtained from SpEx for all the proteins. The value of residuals is defined as the difference between observed and predicted data values, therefore, addressing the quality of the model. We calculated the residuals in the testing set of 990 genes from chr8 for all the models. For the practical analysis, we plotted the density of genes with their associated residual value, which follows Gaussian distribution, satisfying the assumption of the normality of the residuals (Fig. 3). The data is also cross-checked using statistical tests (such as the IFCC-recommended Anderson–Darling test) to ensure it fits a Gaussian distribution. The residual distribution shows the greatest improvement in the RNAPOL II compared to the CTCF and Cohesin (Fig. 3i).

The architectural proteins—CTCF, Cohesin and RNAPOL II, play a diverse role in contributing to gene expression either alone or working together to instruct gene accessibility and expression^57,58^. Therefore, considering that fact, we focused on the residual value of a gene closest to zero by comparing all three proteins named “SpEx-Best”. There is a high density of points close to the origin and a low density of points away from the origin for SpEx-Best compared with the baseline model, which signifies that the gene expression is majorly controlled by the three-dimensional genome structures (Fig. 3i).

To investigate the impact of 3D information on gene expression, we conducted a statistical analysis to determine the mean and standard deviation (SD) of the SpEx-Best residual values which follows the bimodel distribution. We then used this analysis to identify genes that showed the most significant improvement in their expression levels due to incorporating 3D information. Specifically, we considered genes within 0.5 SD of the SpEx-Best distribution, corresponding to a cutoff range of − 1.397 to + 2.106 (Supplementary Fig. 2). We utilised this cutoff to evaluate the efficacy of our model and found that out of 990 genes, 538 were within this range. Among these genes, 363 were found in both models, 168 were specific to SpEx, and only 7 were specific to the baseline model (Fig. 3ii). Our results emphasise the regulatory role of 3D information in gene expression, which is not captured in the baseline model.

Moreover, we assessed the individual impact of each protein on gene expression and observed that their contributions varied. In particular, RNA POL II showed the highest number of improved genes and thus significantly impacted model performance (Fig. 3ii). To further demonstrate the differences, we plotted the value of residuals for each gene for all protein factors and SpEx-Best, highlighting only those genes that fall within the cutoff. We also mapped these highlighted genes (i.e., those within the cutoff of protein factor and SpEx-Best) to the residual of the baseline model (Fig. 3iii). As expected, many genes in the baseline are far from the cutoff and have very high residual values. Therefore we conclude that the proposed model has better efficiency in prediction expression over the baseline model.

**Figure 4:**
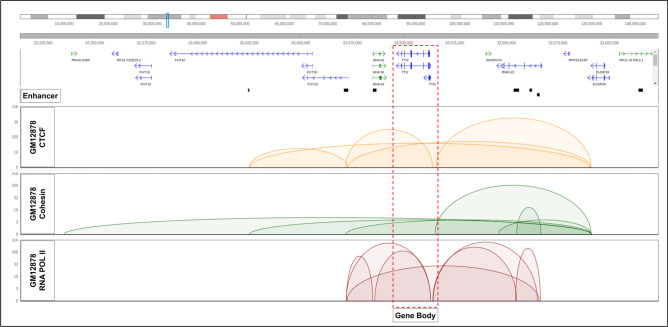
Visualization of the chromosomal region (chr8: 33,320,000–33,625,000) reveals chromatin loops from GM12878 ChIA-PET data, mediated by CTCF (yellow), Cohesin (green), and RNA POLII (red) protein factors. These loops encompass the TTI2 gene locus and interact with a set of enhancers located more than 20 kb away from the transcription start site (TSS). These enhancers, which are not considered in the baseline for gene expression prediction, are taken into account by SpEx, which considers all enhancers within spatial proximity of the transcription start site (TSS) of the gene.

To investigate the improvement of the model, we decided to take a significant example loop in all three datasets—CTCF, Cohesin, and RNA POL II ChIA-PET. The loop was also required to target a gene with an improved prediction score in SpEx over the baseline. The example shows that the gene is spatially close to an enhancer, which plays a crucial role in altering gene expression. For instance, the enhanced prediction score of the expression of the *TTI2* gene in all three protein factors is due to the fact that the *TTI2* gene interacts with subsequent enhancers that are 20 kb apart from the transcription start site but are close enough with the gene in 3D orientation to change the gene expression (Fig. 4).

## Discussion

In this study, we have shown that chromatin's spatial structure significantly influences gene expression. To demonstrate that, we have created an algorithm based on the previous work (ExPecto), and added the processing of the spatial heatmaps created by the ChIA-PET experiments. The experiments were performed using 3 different mediating proteins, thus giving us the maps of the interactions involving those proteins. In all 3 cases, the algorithm improved the baseline model, providing us with up to a 0.042 increase in the Spearman correlation score (such an increase in the case of GM12878 RNAPOL2 ChIA-PET experiment explained an additional 18% of the unexplained part from the baseline model). We have conducted our study on 32 experiments, out of which in 27 we could see improvements. Those findings contribute to the rapid-changing field of three-dimensional genomics, showing that the interactions are indeed required for the proper prediction of the expression—linearly available data, even if we take as many epigenetics factors as in the base ExPecto model (2002), can be still improved with the usage of the spatial data. We have also conducted a case study with *TTI2* gene—an example showed that the model detected spatial proximity of the enhancer, resulting in an increased prediction score. While using multiple factors in the baseline model predicts the expression in a satisfactory way, there are examples where spatial information is significant—as the 20 kbp window might not be enough to fully model the expression level changes. The next step in the field of gene expression prediction is using more modern deep learning architectures—e.g. the ones using transformers, like Enformer—and connecting them with the spatial information for the improvement over the baseline models.

## Conclusions

In conclusion, SpEx extends ExPecto using the spatial information from ChIA-PET experiments, and provides better results on the same datasets compared to the baseline model. The comparisons with the ExPecto and Enformer architectures show that usage of chromatin loop can indeed boost the gene expression prediction scores—as ExPecto obtained an SCC of 0.82, and Enformer 0.85, with the very minor changes to the architecture of ExPecto we were capable of boosting the SCC to 0.83. The usage of the spatial information is definitely worth further investigation—as the ExPecto model already incorporated 2002 epigenetic factors, we firmly believe that the usage of chromatin loops might improve the prediction scores. With the improvement in the machine learning field, we believe that instead of using experimental methods (that we demonstrated to work and improve the quality of the predictions), in-sillico algorithms will be used for the prediction of the contacts, and then those contacts might be used to predict the gene expression.

## Methods

### Obtaining gene expression levels

The gene expression levels were taken from the original ExPecto publication. They have collected and released a file containing expression profiles for 218 tissues (data collected from GTEx, Roadmap epigenomics and ENCODE). We have then manually mapped the ChIA-PET spatial datasets to the closest tissue for which we had an expression profile. The table with mapping can be found in Supplementary Table 1.

### Epigenetic features

The study uses 2002 epigenetic features used in ExPecto paper. What is important, the epigenetic factors include CTCF, RNAPOL2, and cohesin (SMC3) as well—so the model already has information about the epigenetics, and adding the spatial interactions does not yield additional information if the given protein factor is present, or not—that has already been established in the baseline model. Thus, the improvement of the model is not dependent on the existence of the binding factor (e.g. RNAPOL2), but rather on the loop and what is on its other side.

### SpEx architecture

SpEx, as an extension to ExPecto^1^, uses the models described by the authors to generate linear tensors (that are a matrix, where we have 2002 epigenetics features × 10 features showing closeness to the TSS). However, we have added additional spatial information. At the step of generating the final tensors for each gene, an additional spatial tensor is added to the linear one. To create it several steps are executed. First, all the contacts that fall out of the linear scope (20 000 base pairs) are considered. Then, we filter out only the contacts starting or ending near the TSS of the gene, between (TSS, TSS + HiC_resolution), and any other site. Then, only the contacts with a count of at least 2 are considered—which means that in the experiment (be it ChIA-PET or another experiment capable of creating contact matrices), we detected the given contact at least 2 times. Suppose there are no such spacially close regions. In that case, we take instead of them linearly close region again—but to keep the consistency with the spatial organisation, we do not use exponential transformation. After getting the regions to predict, that are spatially close to the TSS in an aforementioned way; the ExPecto prediction is run upon those regions. The predicted signal in the regions is summed to ensure that the tensors are uniform in size. That way, we created the tensors that include not only linear information (< 20,000 bp) but also consider the signal from the regions spatially close to the TSS of the gene. That way, we get a matrix with 2002 epigenetics features × (10 features showing closeness to the TSS + 1 feature representing the regions that are close to TSS in a spatial sense).

The tensors created in that way are saved, as it is computationally expensive to calculate all of them, as both ExPecto and SpEx are calculating them for each of the genes, totalling in 22,827 tensors for each cell line. The second step is an actual prediction of the expression. For that, we have used, as in the ExPecto paper, XGBoost^7^ library. However, we have used different models and parameters. In the case of ExPecto, the model used was GBLinear with reg: linear objective, and we decided to use GBTree with reg:squarederror objective. In the case of SpEx (as the model uses a tree), we have used the tree method of gpu_hist. The full list of parameters used in our model can be found in the code repository.

### Performing the experiments

All the experiments were performed using NVIDIA DGX A100 systems. For each cell line, 22,827 tensors were created using one A100 GPU, 8 CPUs, and 128 GB physical memory. All the tensors took less than 24 h to complete with such settings. Following that, each cell line was subjected to the final training 1000 times to ensure statistical significance of the results, meaning that total 53,000 training were completed (32 cell lines + 21 baselines, without spatial information). In most cases, individual training operations took up to 5 min, and each of the training was assigned one A100 GPU unit, 8 CPUs, and 16 GB of physical memory.

### Statistical analysis of the results

From all the experiments was gathered together, and triple statistical testing was performed for each cell line/factor/tissue. We have used Welch's t-test with independent samples with Bonferroni correction from package statannotations^59^. The results were also tested for the significance in factor-dependent groups (cohesin, CTCF, RNAPOL2) and all together. The residual analysis used an example iteration described in the previous section.

### CTCF and RNAPOL2 datasets

The ChIA-PET CTCF and RNAPOL2 processed data was taken from the 4DNucleome consortium data page (https://data.4dnucleome.org/). The data was obtained there using 4 replicates (2 biological × 2 technical). The pairs were obtained using the ChIA-PIPE^60^ workflow, which produced pairs for each of the replicates. Then, the pairs were merged and processed using a cooler and juicer to obtain the final .mcool files that were downloaded from the database and used in the SpEx algorithm.

### Processing of Cohesin dataset

We gathered the Cohesin ChIA-PET dataset from Encode Portal (https://www.encodeproject.org/) with accession number ENCSR129LGO submitted by Grubert et al. The dataset contains 24 diverse human cell types^47^. We merged the replicates and then processed them with the ChIA-PIPE pipeline^60^ using the default parameters (Linker Sequence = GTTGGATAAG and Peak-calling Algorithm = MACS2). The pipeline generated a high-resolution 2D contact matrix (in .hic file format) along with the annotated chromatin loops with their binding peak overlap. These .hic files were then converted into .mcools files using the hic2cool tool (https://github.com/4dn-dcic/hic2cool) developed by 4DNucleome to obtain the final input for the SpEx algorithm.

### Division of the data into training and testing sets

All the cell lines and baseline models were processed uniformly to create training and testing sets. Chromosomes X and Y were excluded from the study, and then all chromosomes except chromosome 8 were taken into the training set, and chromosome 8 was used exclusively for testing purposes. That way, we ensured that the testing data was not used in any way during the training. Chromosome 8 was taken as one of the chromosomes close to the mean size, as well as to compare our study to the original ExPecto paper—as they have used the same setup.

## Supplementary Information

Supplementary Information.

## Data Availability

The algorithm is available at https://github.com/SFGLab/spex/. The data used for the experiments is available at https://data.4dnucleome.org/ and https://www.encodeproject.org/ and the precise accession numbers are provided in the Supplementary Files.
