# Supplementary material for: Enhanced performance of gene expression predictive models with protein-mediated spatial chromatin interactions
Source: Sci Rep. 2023 Jul 20;13:11693. doi: 10.1038/s41598-023-38865-5 (PMC10359366; doi:10.1038/s41598-023-38865-5)
Supplement: Supplementary file 1 — Supplementary Information. [file 41598_2023_38865_MOESM1_ESM.docx]

Supplementary information

[**Supplementary Figure 1.** Statistical analysis of the spearman correlation score between the baseline (no 3D information), and with the cell line specific heatmaps available for the model.](#_2d5plh2m9b0n) **2**

[**Supplementary Figure 2.** (A) Distribution of Residual value of gene in SpEx and Baseline. (B) Standard deviation (SD) of Gaussian distribution of residual value, highlingth significant difference of 0.5 SD for Baseline and SpEx (best value) (C ) Distribution of Residual value of gene between the baselines, and the experiments grouped by the factor of interest (CTCF, Cohesin and RNAPOL2). (D) Standard deviation (SD) of Gaussian distribution of residual value, highlingth significant difference of 0.5 SD for all three proteins.](#_95vrgdahoh3q) **6**

[**Supplementary Figure 3.** Statistical analysis of the pearson correlation coefficient between the baselines and the experiments grouped by the factor of interest (cohesin, CTCF, RNAPOL2).](#_yip2hasg1i7l) **7**

[**Supplementary Figure 4.** Statistical analysis of the pearson correlation coefficient between the baseline (no 3D information), and with the cell line specific heatmaps available for the model.](#_azyv04hso9h) **8**

[**Supplementary Figure 5.** Statistical analysis of the Root-mean-square error (RMSE) between the baselines and the experiments grouped by the factor of interest (cohesin, CTCF, RNAPOL2).](#_csnkmqw55aqn) **12**

[**Supplementary Figure 6.** Statistical analysis of the Root-mean-square error (RMSE) between the baseline (no 3D information), and with the cell line specific heatmaps available for the model.](#_yhih2hh5z4di) **13**

[**Supplementary Table 1.** Accession numbers of the CTCF, RNAPOL2, and cohesin ChIA-PET data used in the study, along with the mapping of the ChIA-PET datasets to the tissue expression profile.](#_s77rt32ht77t) **17**

###### **Supplementary Figure 1.** Statistical analysis of the spearman correlation score between the baseline (no 3D information), and with the cell line specific heatmaps available for the model.
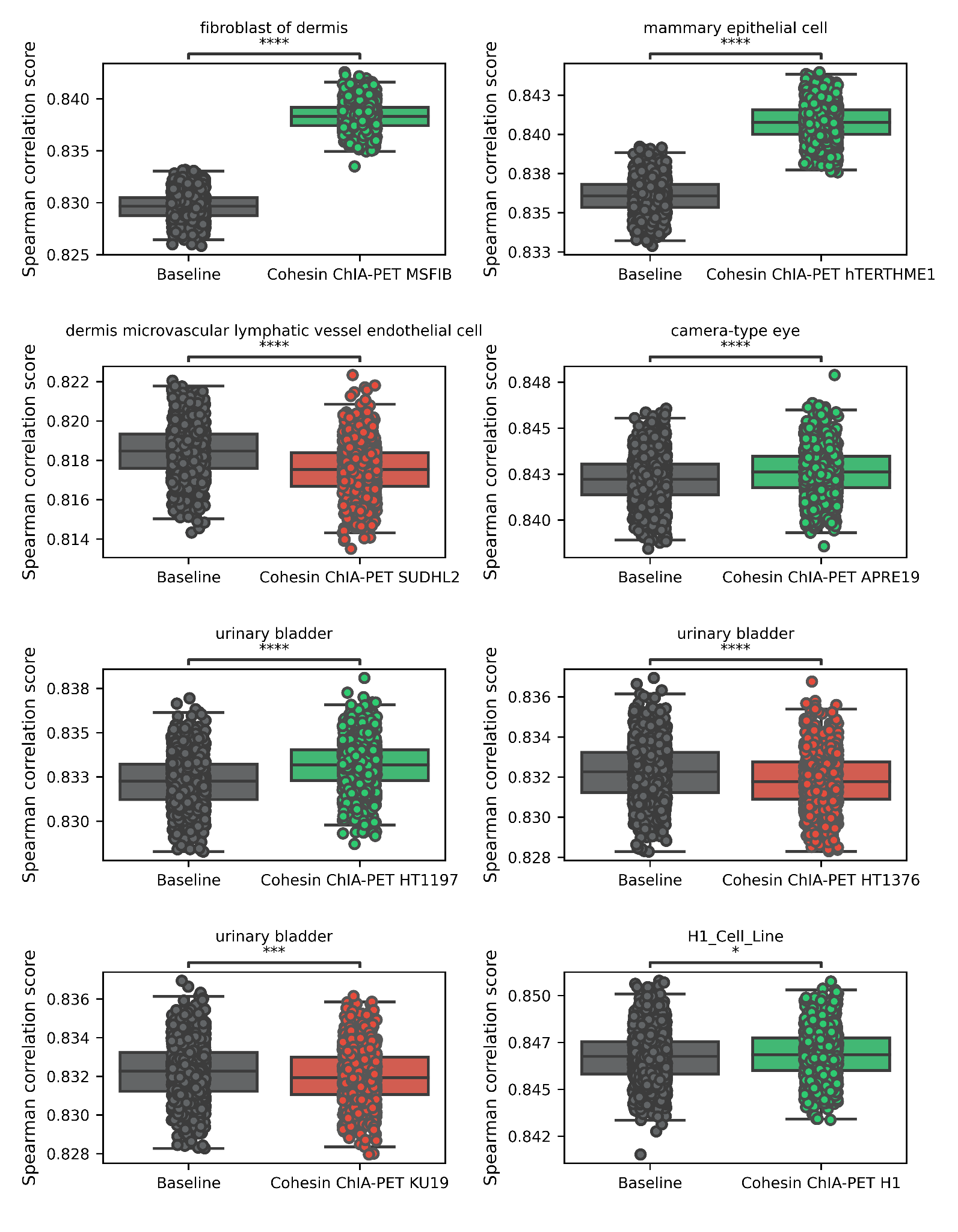

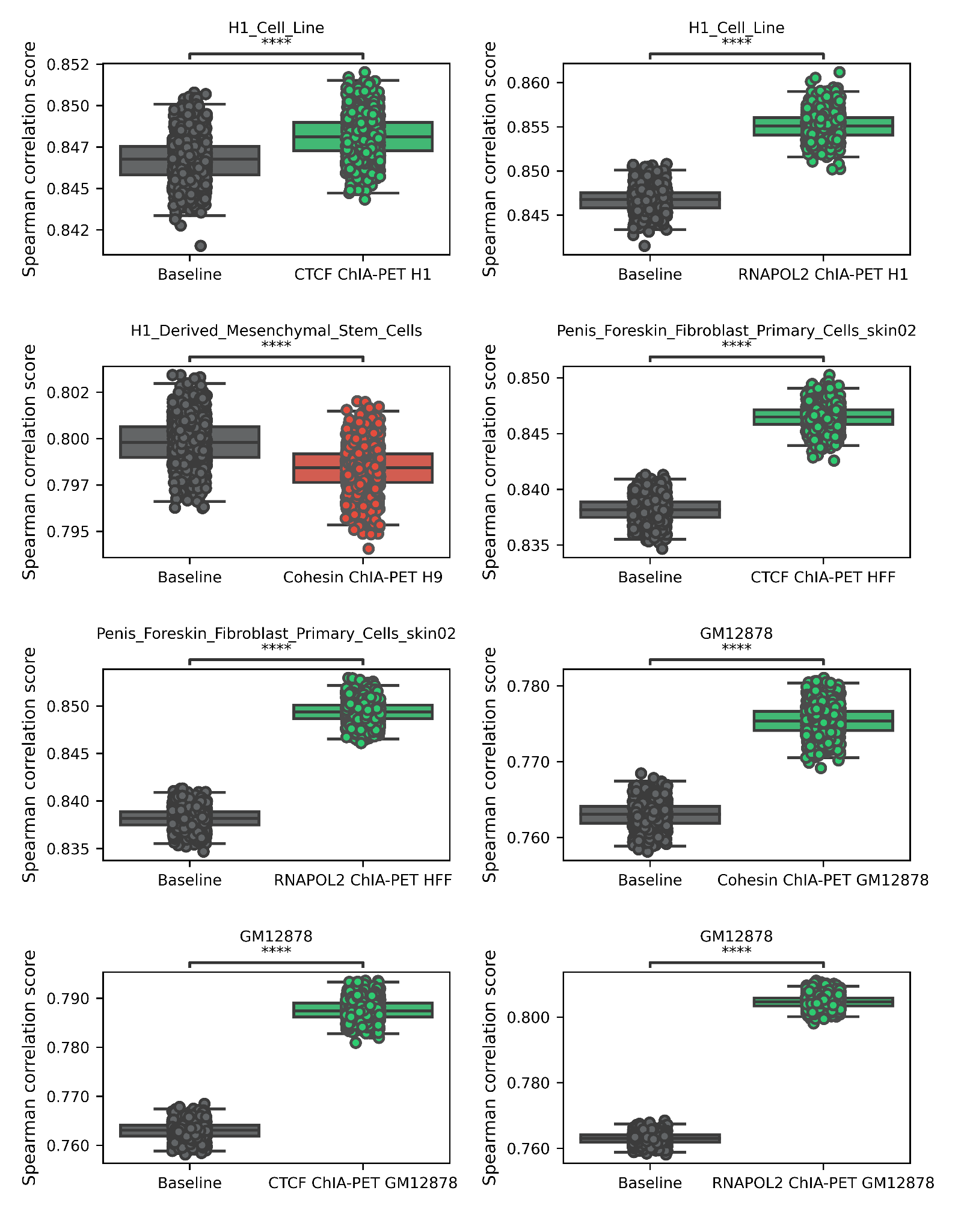

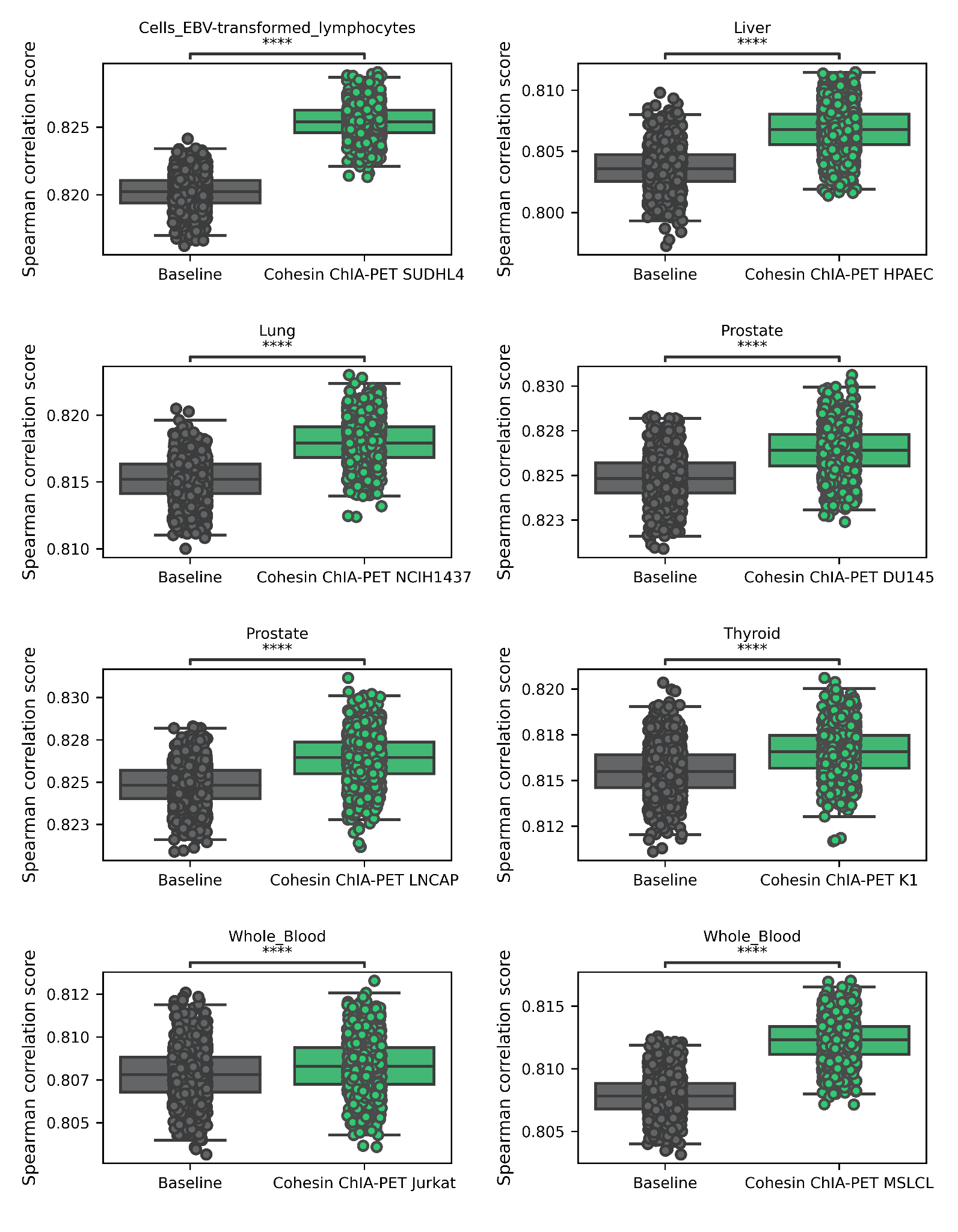

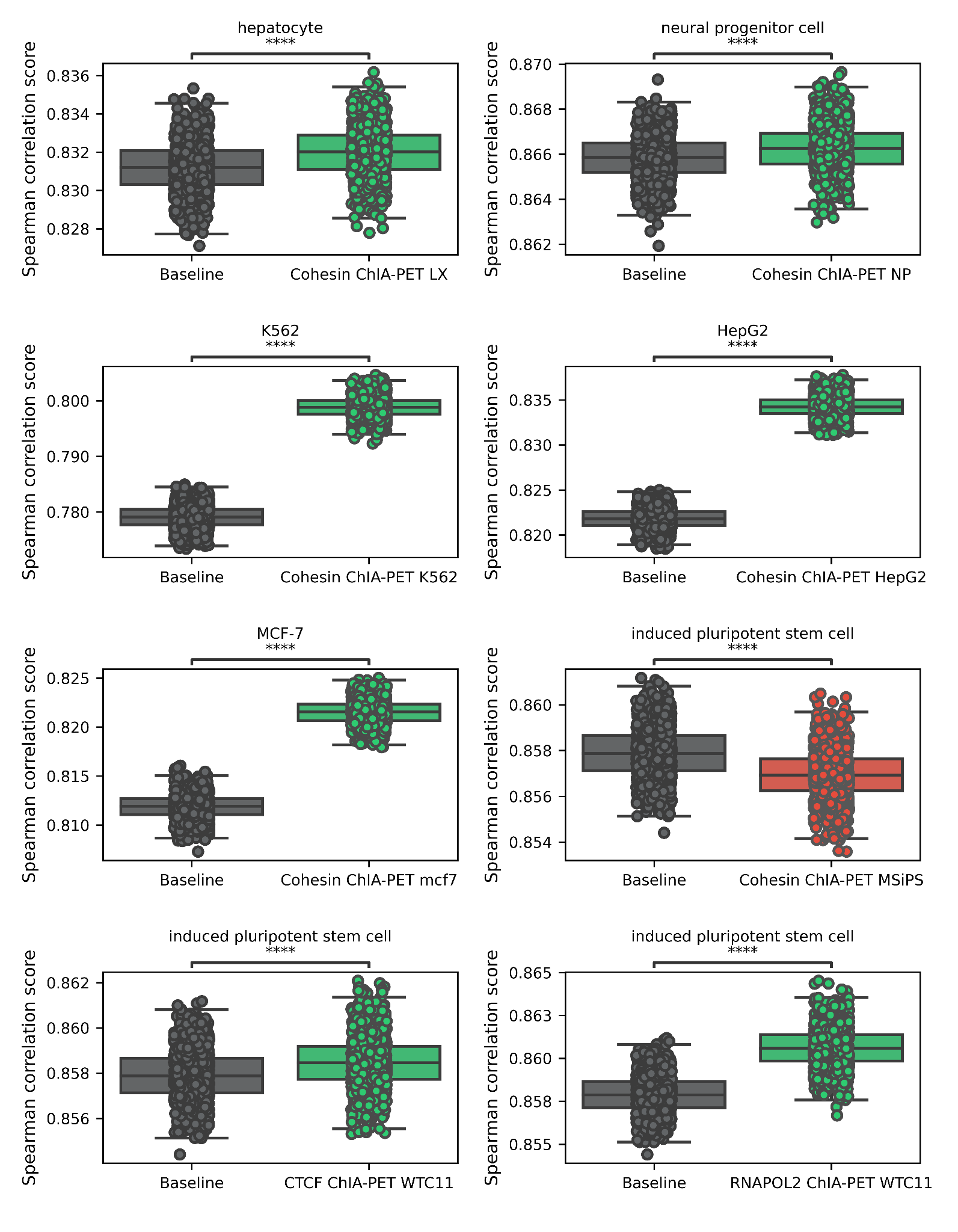


###### **Supplementary Figure 2**. (A) Distribution of Residual value of gene in SpEx and Baseline. (B) Cutoff calculated on the bimodal distribution of residual value, highlighted with significant difference on Baseline and SpEx (best value) (C) Distribution of Residual value of gene between the baselines, and the experiments grouped by the factor of interest (CTCF, Cohesin and RNAPOL2). (D) Cutoff calculated on the bimodal distribution of residual value, highlingth significant difference for all three proteins.


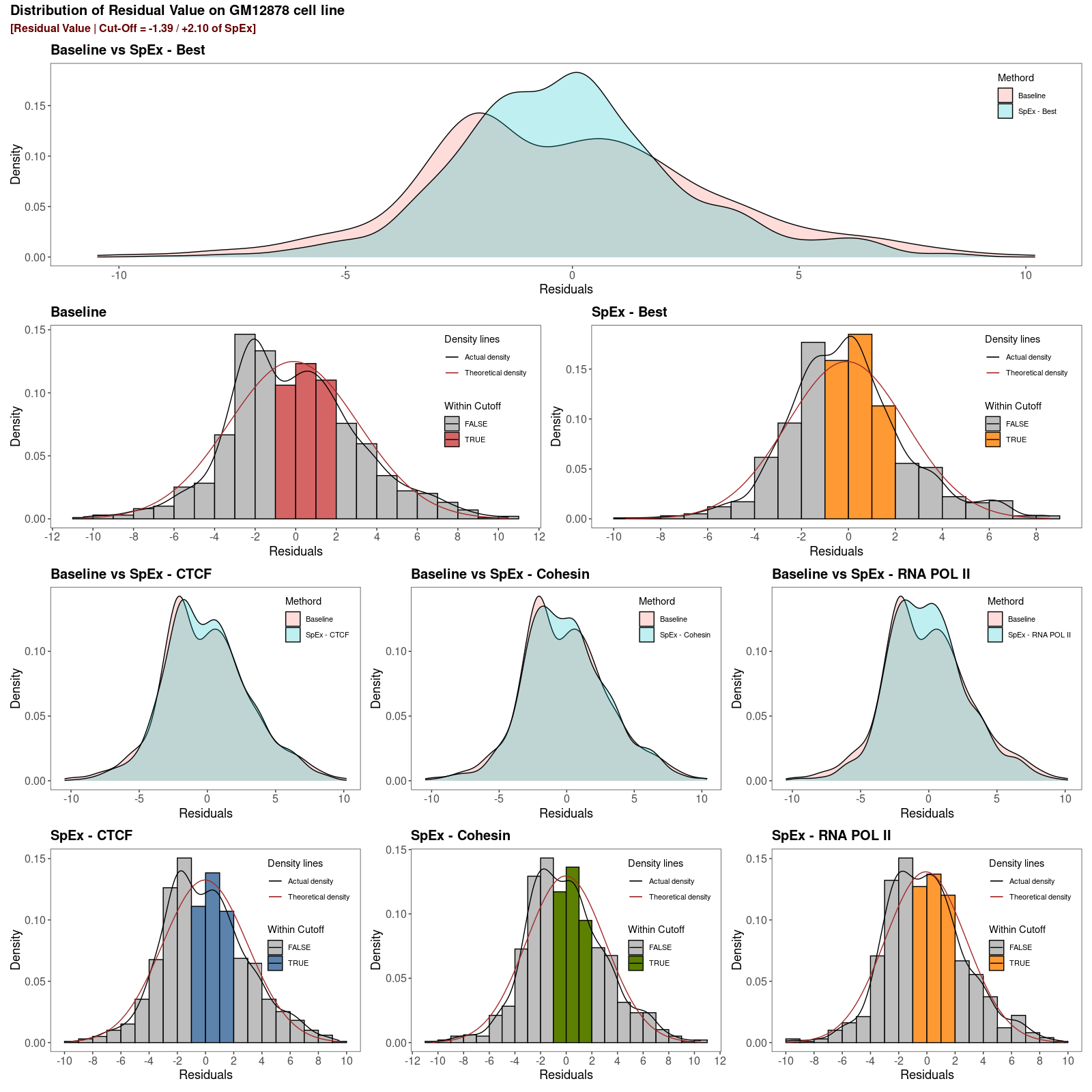


###### **Supplementary Figure 3.** Statistical analysis of the pearson correlation coefficient between the baselines and the experiments grouped by the factor of interest (cohesin, CTCF, RNAPOL2).
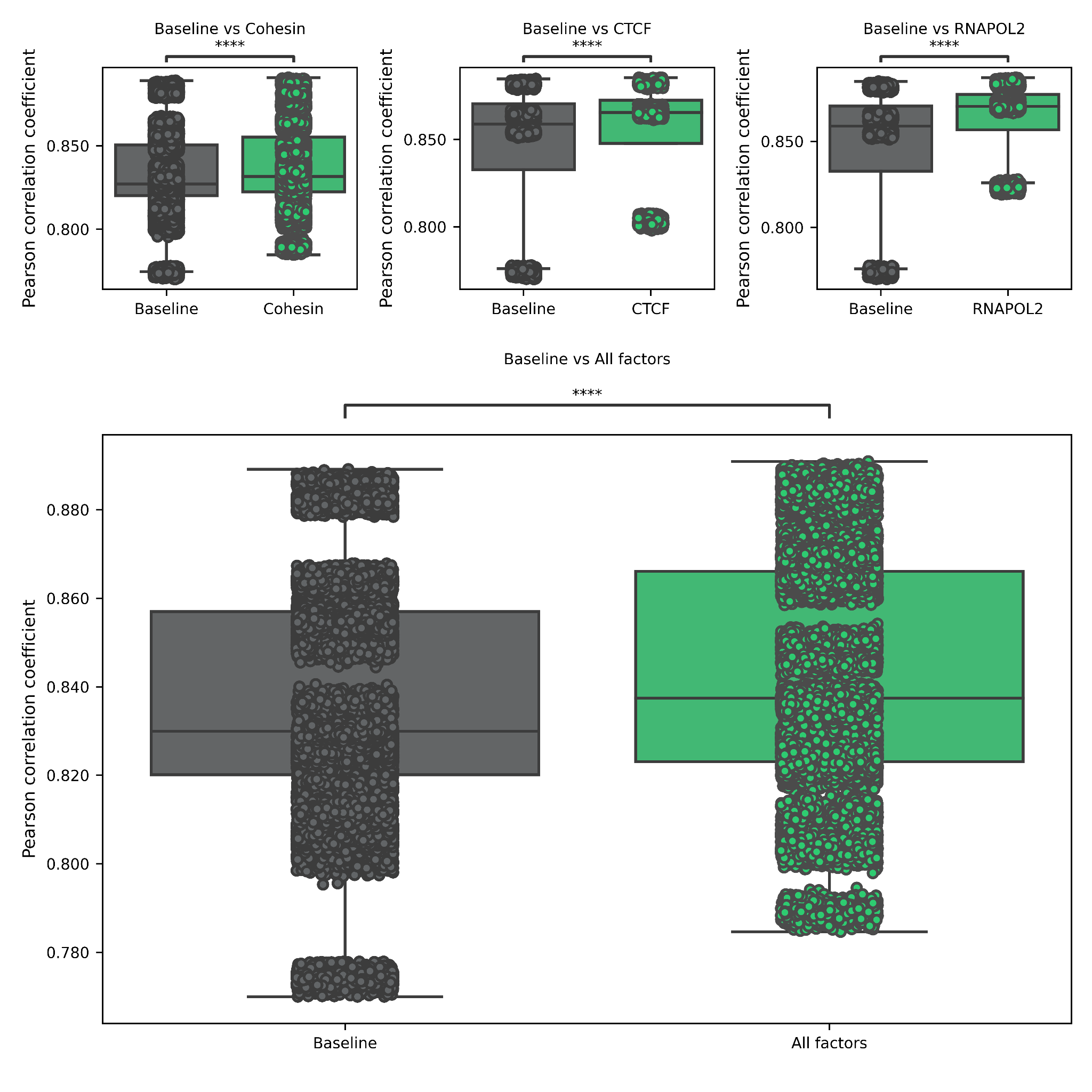


###### **Supplementary Figure 4.** Statistical analysis of the pearson correlation coefficient between the baseline (no 3D information), and with the cell line specific heatmaps available for the model.
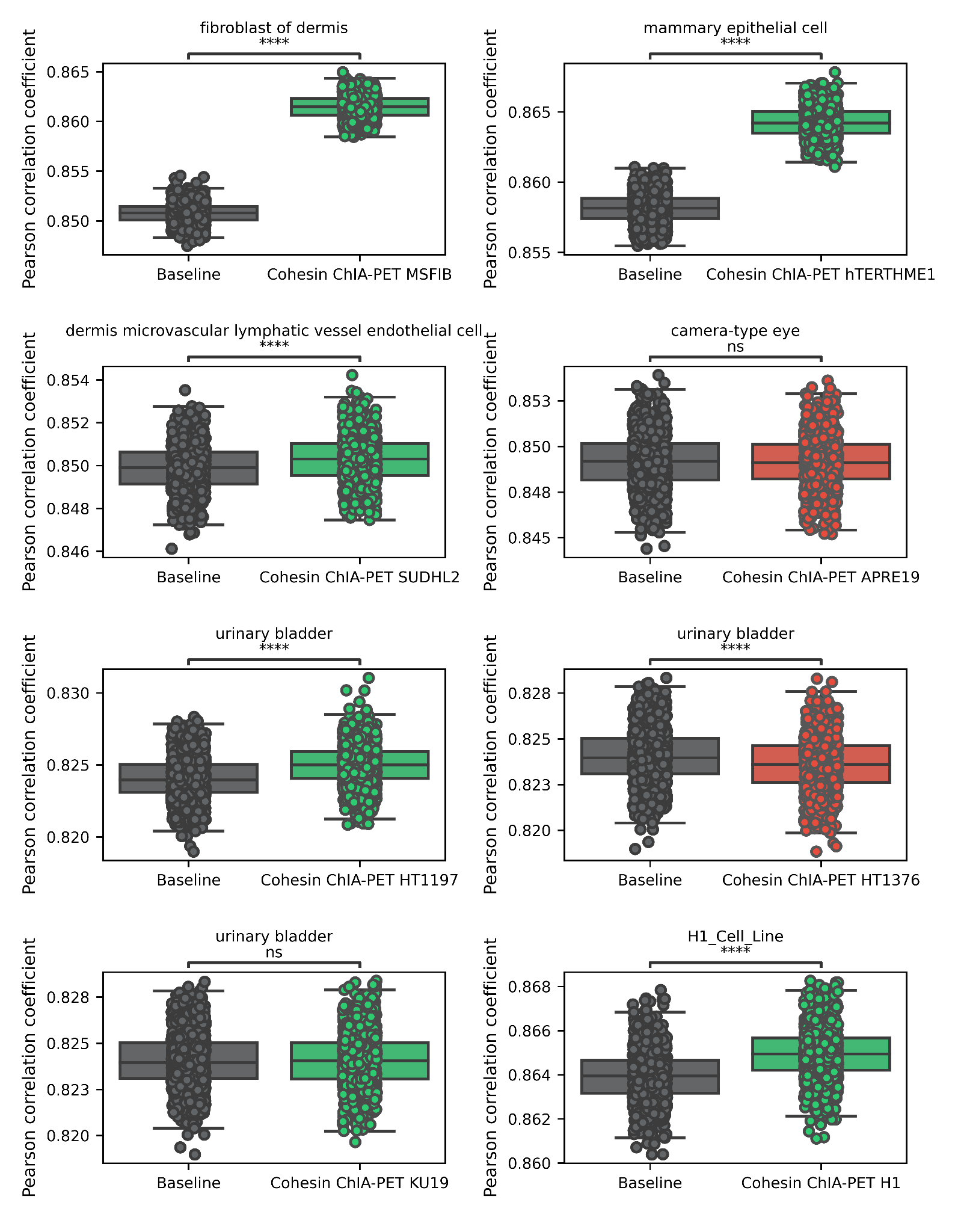

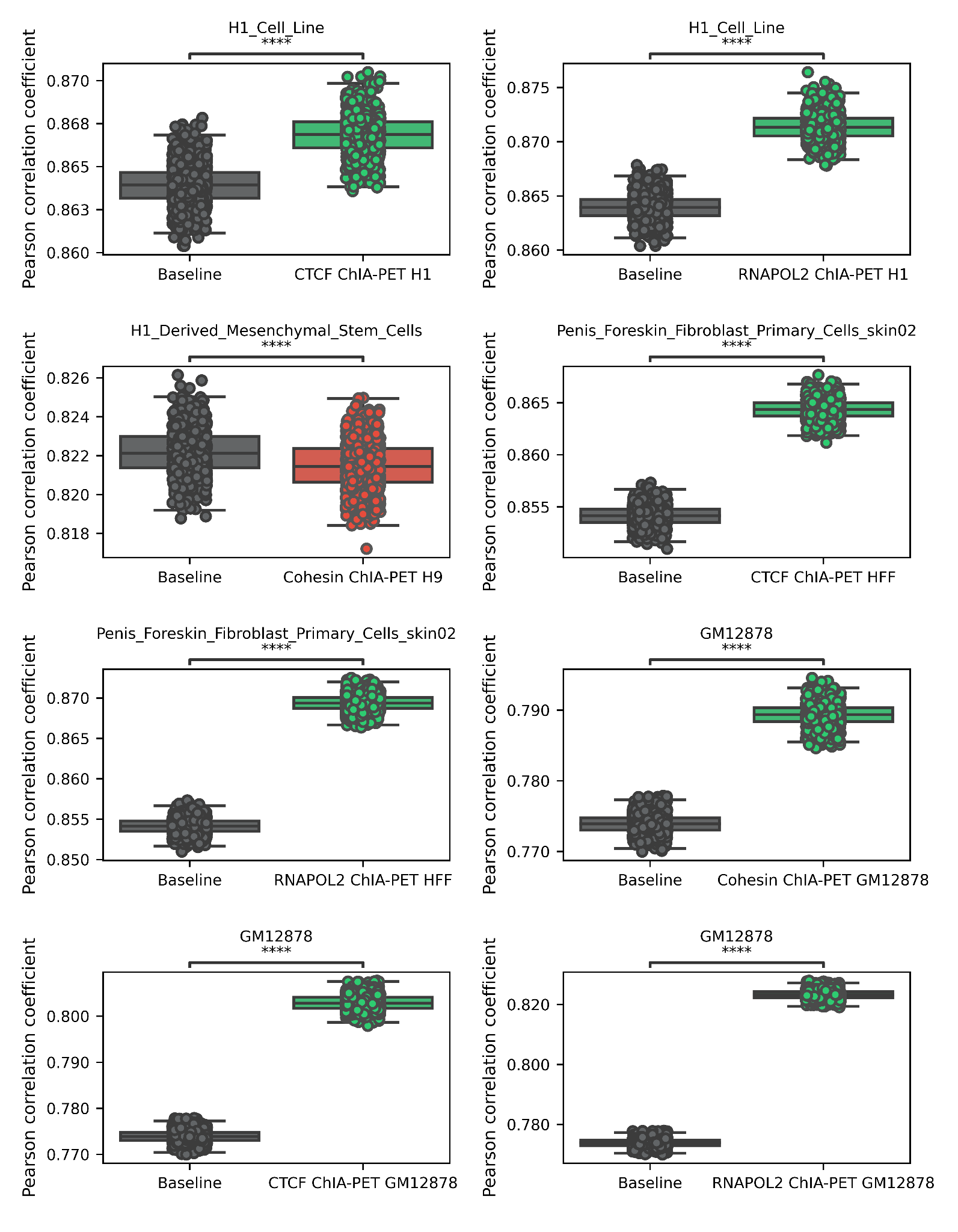

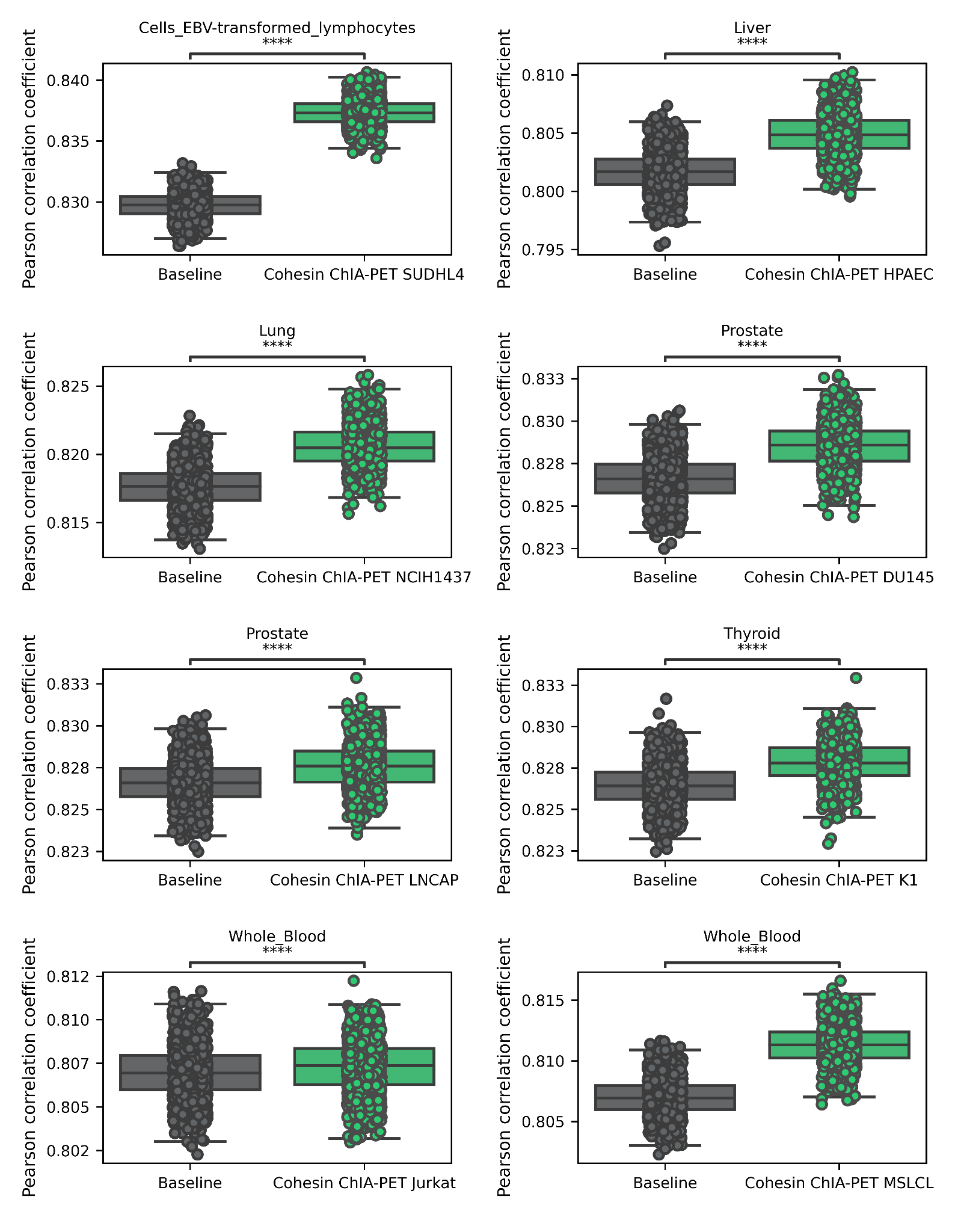

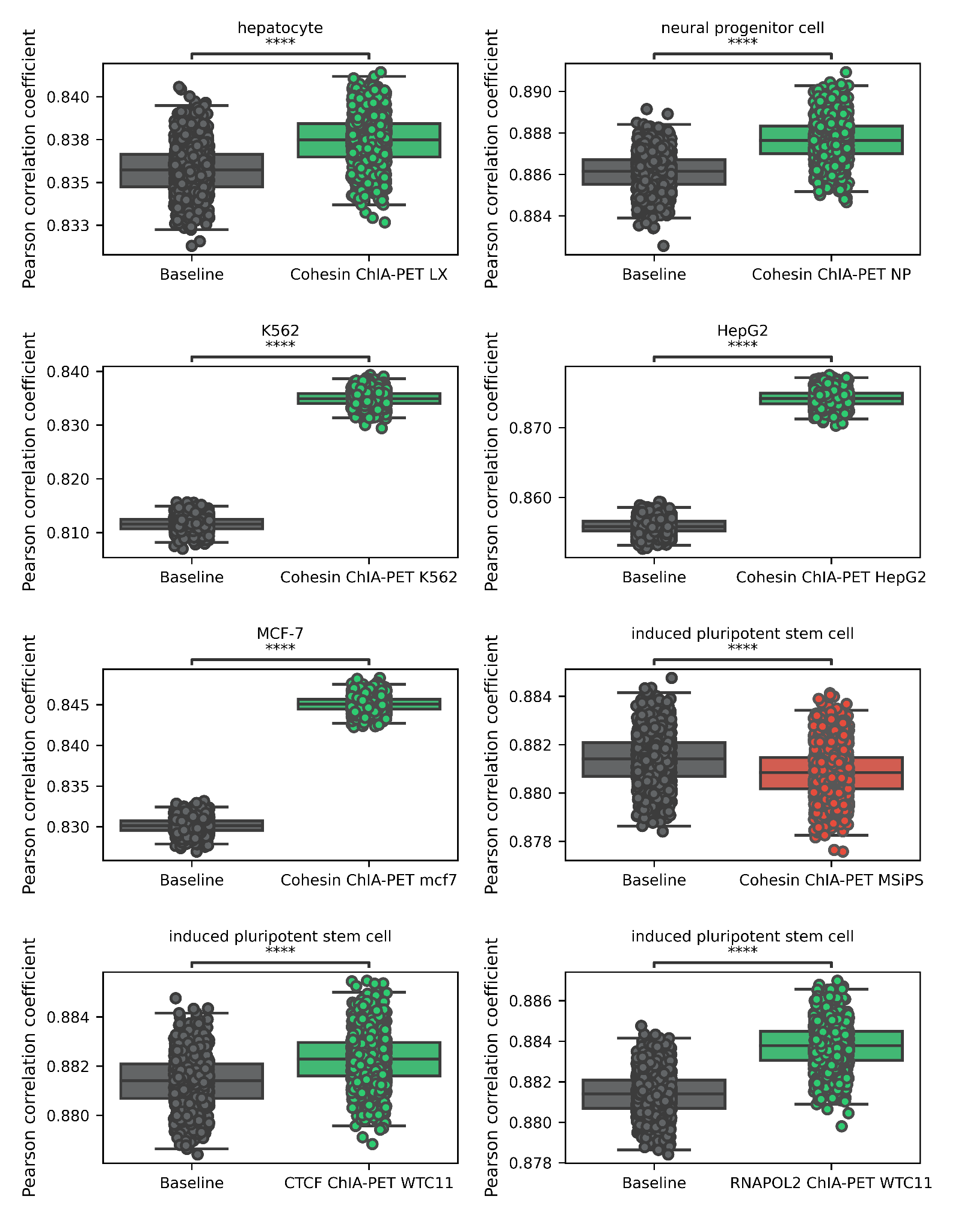


###### **Supplementary Figure 5.** Statistical analysis of the Root-mean-square error (RMSE) between the baselines and the experiments grouped by the factor of interest (cohesin, CTCF, RNAPOL2).**
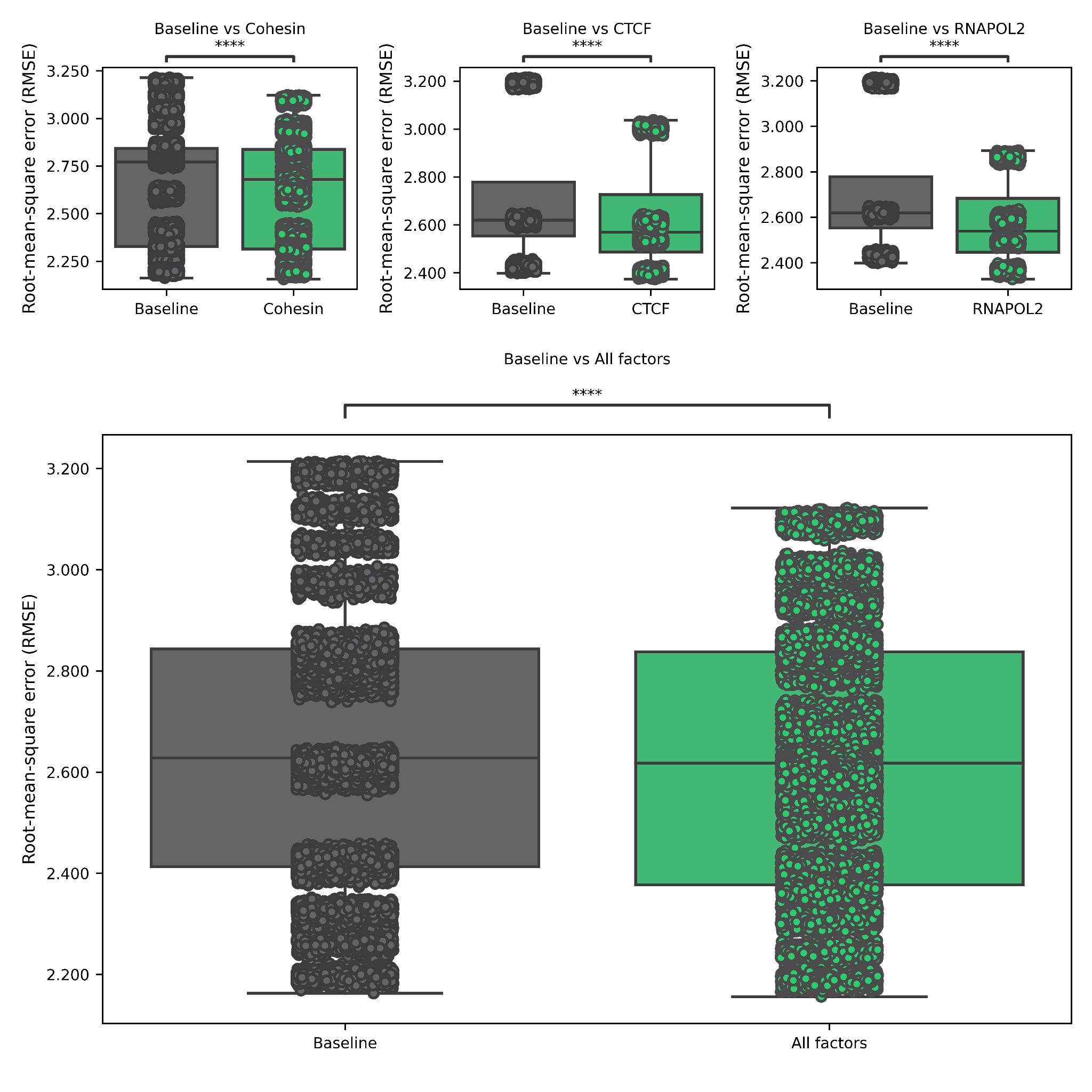
**

###### **Supplementary Figure 6.** Statistical analysis of the Root-mean-square error (RMSE) between the baseline (no 3D information), and with the cell line specific heatmaps available for the model.**
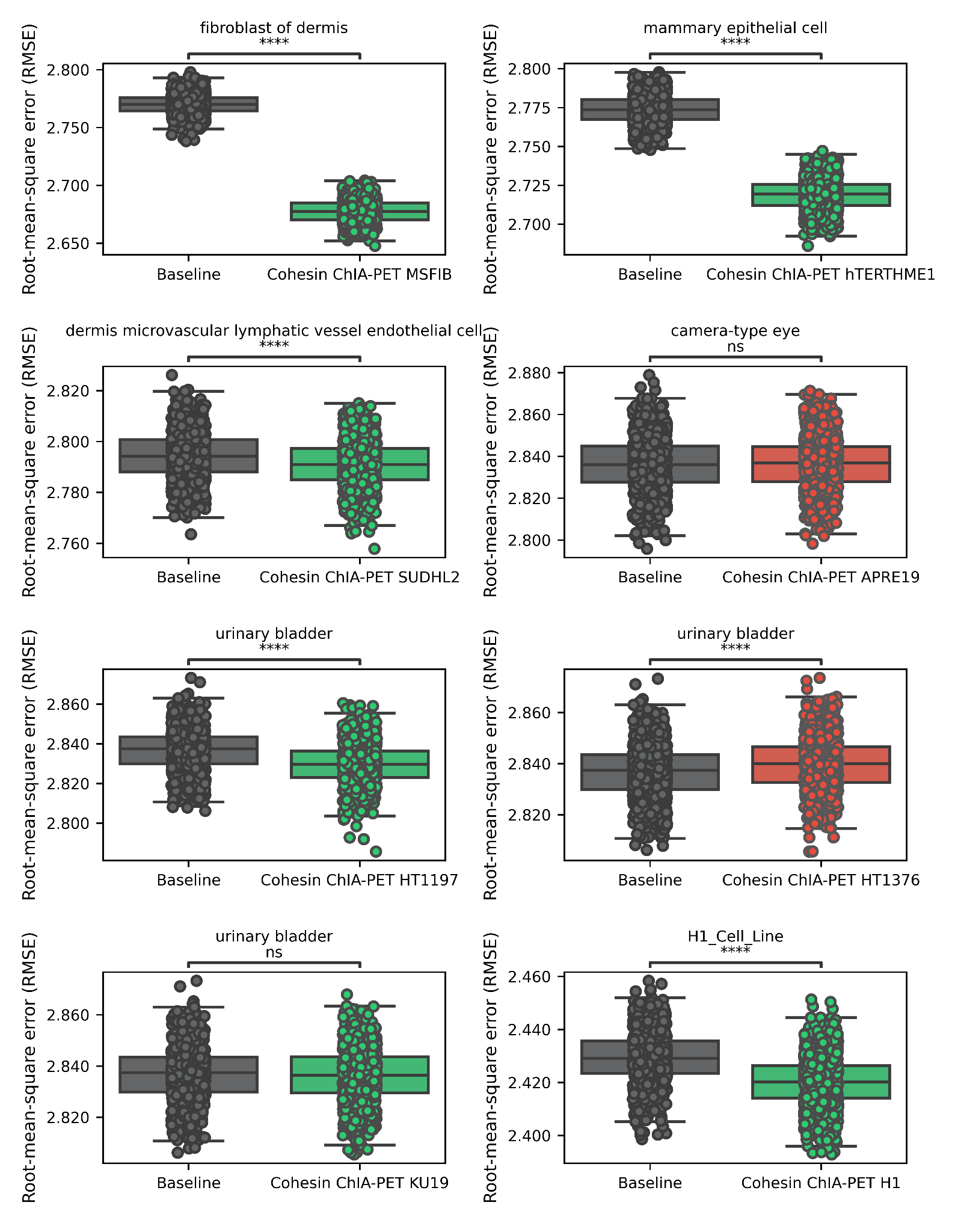

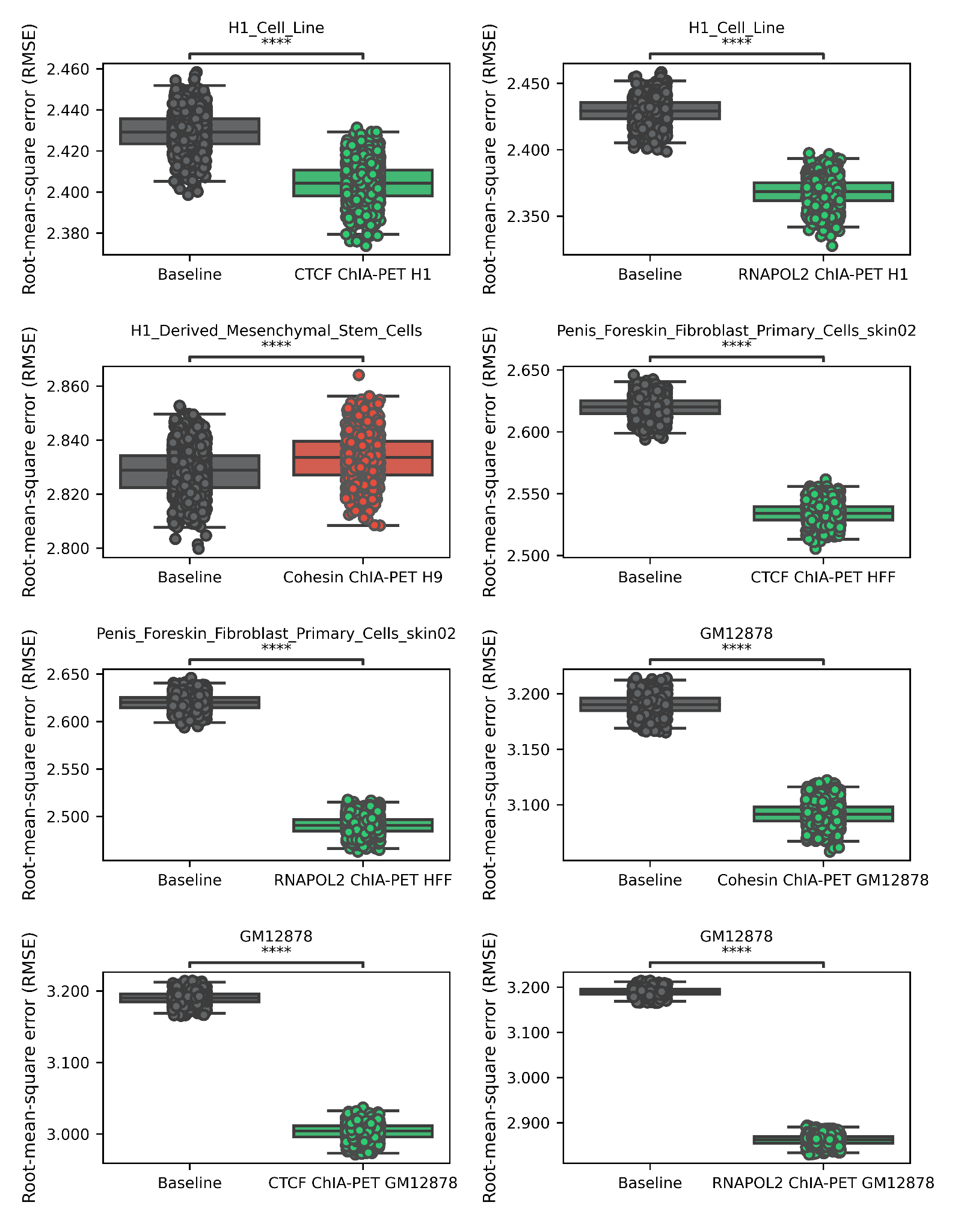

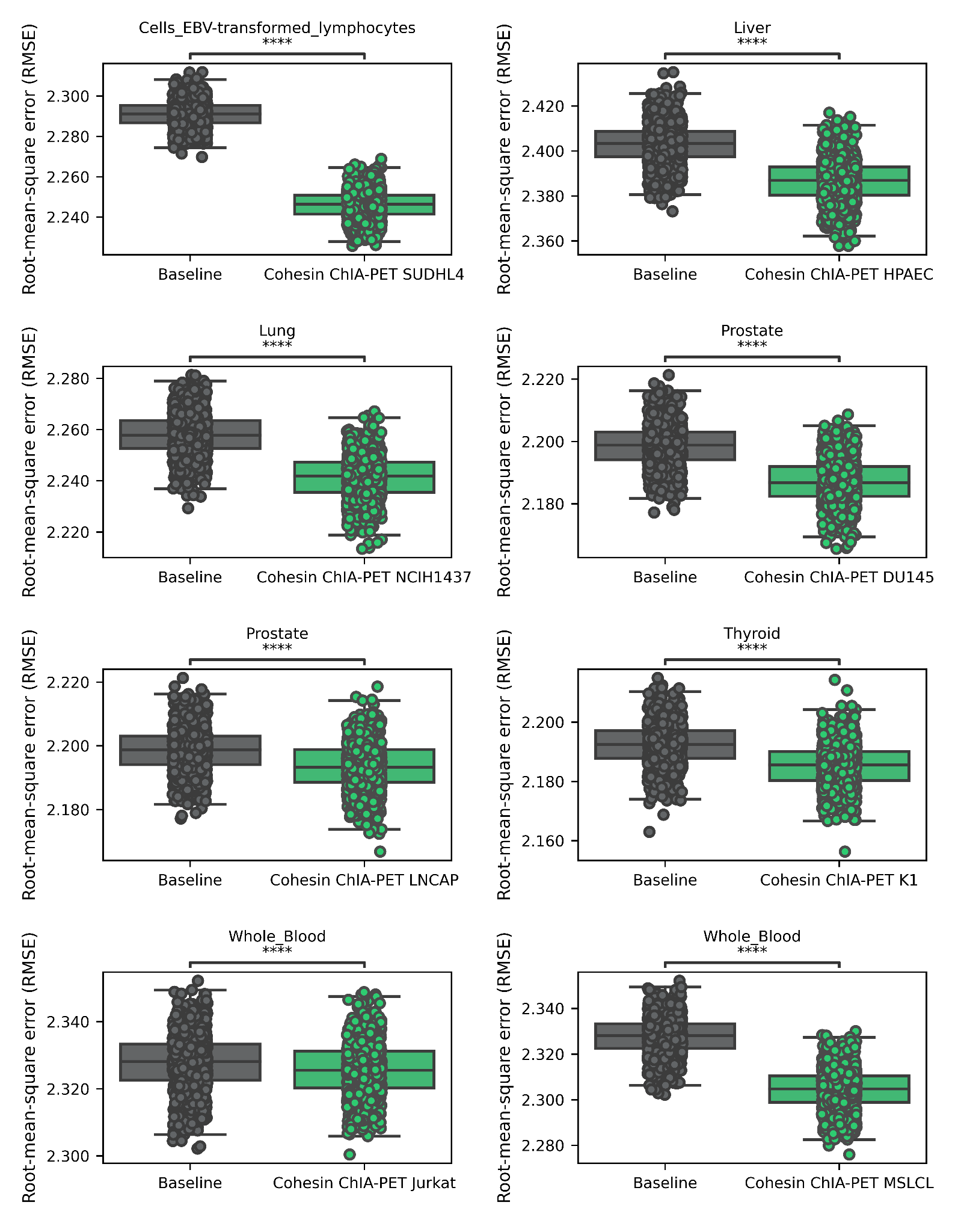

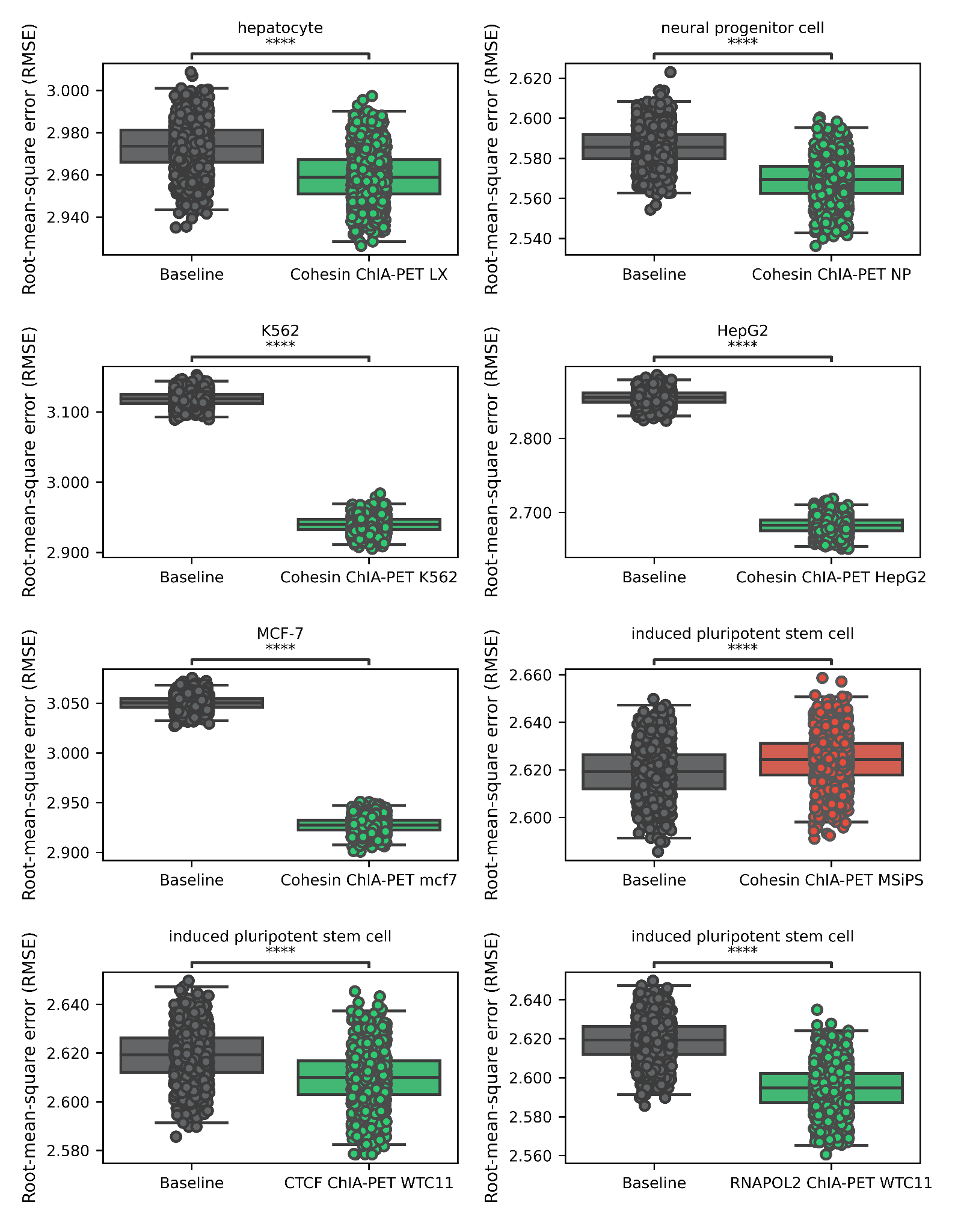
**

###### Supplementary Table 1. Accession numbers of the CTCF, RNAPOL2, and cohesin ChIA-PET data used in the study, along with the mapping of the ChIA-PET datasets to the tissue expression profile.

| **CTCF ChIA-PET** | | |
| --- | --- | --- |
| **Cell Line** | **Accession Number (4DNucleome)** | **Mapped expression profile** |
| GM12878 | 4DNFIUEG39YZ | GM12878 |
| H1 | 4DNFIK3276U7 | H1_Cell_Line |
| HFFc6 | 4DNFIELOAD41 | Penis_Foreskin_Fibroblast_Primary_Cells_skin02 |
| WTC11 | 4DNFI9DV93LN | induced pluripotent stem cell |

| **RNAPOL II ChIA-PET** | | |
| --- | --- | --- |
| **Cell Line** | **Accession Number (4DNucleome)** | **Mapped expression profile** |
| GM12878 | 4DNFIXG4BEEL | GM12878 |
| H1 | 4DNFIF1J6GC5 | H1_Cell_Line |
| HFFc6 | 4DNFIOOSHTQV | Penis_Foreskin_Fibroblast_Primary_Cells_skin02 |
| WTC11 | 4DNFIMJXO26D | induced pluripotent stem cell |

| **Cohesin ChIA-PET** | | |
| --- | --- | --- |
| **Cell line** | **Accession Number (ENCODE)** | **Mapped expression profile** |
| HepG2 | ENCSR146FPM | HepG2 |
| ARPE-19 | ENCSR110JOO | camera-type eye |
| Jurkat | ENCSR361AYD | Whole_Blood |
| MCF7 | ENCSR255XYX | MCF-7 |
| DU 145 | ENCSR672RHL | Prostate |
| NCI-H1437 | ENCSR833CMG | Lung |
| HT-1376 | ENCSR933UZH | urinary bladder |
| MSLCL | ENCSR452NHL | Whole_Blood |
| SU-DHL-2 | ENCSR128RPG | dermis microvascular lymphatic vessel endothelial cell |
| SU-DHL-4 | ENCSR658RQQ | Cells_EBV-transformed_lymphocytes |
| GM12878 | ENCSR981FNA | GM12878 |
| LNCaP | ENCSR113OIR | Prostate |
| HPAEC | ENCSR636PMP | Liver |
| MSFIB | ENCSR732QOH | fibroblast of dermis |
| K1 | ENCSR314HAC | Thyroid |
| H1-hESC | ENCSR543YTV | H1_Cell_Line |
| hTERT-HME1 | ENCSR991JXX | mammary epithelial cell |
| MSiPS | ENCSR778FXH | induced pluripotent stem cell |
| LX | ENCSR381DCY | hepatocyte |
| NP | ENCSR247RGI | neural progenitor cell |
| H9-hESC | ENCSR478BMT | H1_Derived_Mesenchymal_Stem_Cells |
| KU19 | ENCSR404HWQ | urinary bladder |
| K562 | ENCSR338WUS | K562 |
| HT-1197 | ENCSR386KHY | urinary bladder |

###### 
